# Unmasking Distress: An Analysis of COVID-19’s Mental Health Impact on Nurses in South Africa

**DOI:** 10.1177/01939459251316049

**Published:** 2025-02-03

**Authors:** Phillipa Haine, Ashraf Kagee, Bronwyne Coetzee, Marnus Janse Van Vuuren, Lindokuhle Shongwe

**Affiliations:** 1 Department of Psychology, Stellenbosch University, Stellenbosch, Cape Town, South Africa

**Keywords:** alcohol consumption, anxiety, COVID-19, depression, nurses, post-traumatic stress

## Abstract

**Background:**

The acute mental health challenges faced by nurses during the COVID-19 pandemic have the potential to result in long-lasting psychological impacts. Promoting the mental health of nurses is crucial not only to safeguard their wellbeing but also to ensure the delivery of optimal patient care.

**Objective:**

This study sought to ascertain the extended mental health impact of the COVID-19 pandemic among nurses in South Africa.

**Methods:**

Participants involved nurses working at four hospitals in the Western Cape province: Helderberg, TC Newman, Stellenbosch, and Tygerberg. A convenience sample of nurses (*N* = 264) from the four hospitals participated in the study. Data collection involved an online survey, which included a comprehensive battery of psychological measures, such as fear of COVID-19, perceived vulnerability to disease, anxiety, depression, post-traumatic stress disorder (PTSD), alcohol use, and worry about COVID-19 infection.

**Results:**

The mean age of participants was 34.4 (*SD* = 7.9), with a majority being female (82%). Age was positively correlated with hopelessness and life satisfaction but negatively associated with fear of COVID-19 and symptoms of anxiety, PTSD, alcohol use, and depression. Nurses in the private sector reported higher levels of alcohol use and PTSD than nurses in public, while married and partnered nurses reported reduced levels of anxiety compared to their unmarried counterparts. Higher education levels were associated with reduced adverse mental health outcomes.

**Conclusion:**

Psychological distress among nurses was exacerbated even after the peak of the COVID-19 pandemic. Potential areas of concern are highlighted, indicating the need for targeted interventions.

The COVID-19 pandemic has engendered socio-economic circumstances that have accentuated mental health issues, emotional distress, and deleterious substance utilization in populations across the globe.^1,2^ Healthcare workers (HWCs), especially those in low- and middle-income countries,^3,4^ confront a myriad of challenges which exacerbate their vulnerability to adverse psychological outcomes.^5^ Within South Africa, for example, HCWs face significant challenges that predate the pandemic, including high disease burdens such as the human immunodeficiency virus and tuberculosis, insufficient human capital, poor leadership and management, and disparities in resource allocation across provinces and between the public and private healthcare sectors.^6–8^ The unique socio-economic and healthcare landscape of South Africa, coupled with the unprecedented challenges posed by the pandemic, place the mental well-being of HCWs in South Africa in a precarious position, necessitating a more focused investigation.

Nurses, comprising a large majority of the healthcare workforce^9^ and uniquely distinguished by their tireless presence at the bedsides of patients, have consistently demonstrated elevated levels of occupational stress and consequent distress, surpassing counterparts in other occupational groups in comparable infectious disease outbreaks.^10,11^ Nurses often experience burnout given the inherently demanding nature of the profession.^12^ Compounding factors impacting the mental health of nurses during the pandemic include a surge in critically ill patients, extended working hours, a heightened risk and fear of virus infection,^13^ the emotional toll of patient and colleague suffering and death, as well as shortages in personal protective equipment and other medical resources.^9,14–16^ In addition, feelings of uncertainty as well as evolving healthcare protocols heighten nurses’ stress, while physical distancing requirements exacerbate feelings of social isolation and a lack of peer support, all of which are critical in managing stress. Moreover, nursing staff may grapple with moral distress when confronted with challenging triage decisions^17^ and encounter societal stigma, which includes being perceived as a threat to others and labeled as “disease-carriers.”^17^(p.2743)

The psychological consequences of COVID-19 among nurses in South Africa remain under-researched, with studies predominantly focusing on HCWs as a broad group.^18,19^ Notable exceptions include work by Engelbrecht et al., Kelly et al., Onwubu et al., and Coetzee et al.^20–23^ While Engelbrecht et al. examined post-traumatic stress disorder (PTSD) and coping strategies among nurses (n = 286) in the Free State province,^20^ Kelly et al. qualitatively explored the reflections of nurses (n = 13) working in public health facilities in the Eastern Cape province.^21^ Similarly, Onwubu et al. qualitatively explored the experiences of public sector nurses in providing mental healthcare services during the COVID-19 pandemic.^22^ Comparably, Coetzee et al. investigate the protective role of satisfaction with life, sense of coherence, and resilience in the relationship between depression, social support, fear of COVID-19, and perceived vulnerability to disease among nurses (n = 264) in the Western Cape province.^23^ Engelbrecht et al. found that more than 4 in every 10 nurses screened positive for high levels of post-traumatic stress and called for targeted interventions providing nursing staff with healthy coping mechanisms.^20^ Similarly, Kelly et al. illuminated the daily challenges facing nurses amid the pandemic, including equipment and staff shortages, which significantly impacted their overall well-being and service delivery.^21^ Likewise, Onwubu et al. demonstrated that existing challenges were exacerbated for nurses, including reduced patient visits, complexities in healthcare provision, and a lack of adequate support.^22^ Notably, Coetzee et al. found that higher levels of fear of COVID-19 strongly predicted depressive symptoms among nurses, with a sense of coherence and social support emerging as robust protective resources.^23^

The limited data on the mental health ramifications of the COVID-19 pandemic among nurses as a unique group in South Africa is significant given a recent systematic review and meta-analysis indicating heightened levels of psychological distress and mental health disorders among nurses compared to other categories of HCWs.^24^ While a scoping review of the literature found that poor psychological outcomes among nurses predominantly involve elevated levels of burnout, depression, anxiety, PTSD, sleep disorders, low quality of life, and fear of infection and death,^9^ a systematic review and meta-analysis found pooled prevalence rates among nurses to be 43% for stress, 37% for anxiety, 35% for depression and 43% for insomnia.^25^ Recent studies further point to harmful alcohol use as a concern among nurses amid the pandemic. Among a sample of 57 nurses in the United States, for example, more than one-third of nurses reported risky or harmful alcohol use.^26^

Several studies have highlighted that the acute mental health challenges experienced by HCWs can have long-term psychological consequences.^27^ For example, research by Lee et al. found that HCWs continued to experience significantly elevated levels of stress, depression, anxiety, and posttraumatic symptoms even 1 year after the SARS outbreak, in comparison to non-healthcare professionals.^28^ Similarly, a study of 5223 nurses and social workers in the United Kingdom reported substantial deterioration in mental health across two pandemic time points: Phase 1 (May–July 2020) and Phase 2 (November 2020–January 2021).^29^ The marked decline in nurses’ mental health over time raises serious concerns, both for the healthcare profession and the patients whom they serve. Examining the mental health challenges faced by nurses in South Africa, even after the immediate threat of the pandemic has subsided, is thus essential.

## Worry About COVID-19

Worry is a cognitive process involving persistent, often uncontrollable thoughts about uncertain or potentially negative future events.^30^ While some worry can be adaptive, excessive worry has been shown to contribute to psychological distress.^30,31^ Psychological distress, as described by Mirowsky and Ross, refers to “a state of emotional suffering characterized by symptoms of depression and anxiety, which are often linked to stressful life events or circumstances.”^31^(p.10)

Data from several studies emphasize that nurses worry about becoming infected or infecting loved ones with COVID-19.^32,33^ Investigations into COVID-19-related worries in nurses are critical given literature demonstrating associations between worry and increased risk of burnout, psychological distress, and risk of psychiatric morbidity among HCWs.^34,35^ Among a large sample of nurses surveyed over time in Italy, for example, Sampaio et al.^34^ found that the only factors positively associated with nurses’ symptoms of depression, anxiety, and stress were concerns about infecting others and worries about becoming infected. Comparably, among a sample of 96 intensive care unit professionals surveyed in the United Kingdom, Vandevala et al.^35^ demonstrated that rumination was positively associated with burnout, depression, and risk of psychiatric morbidity.

Galletta et al.^32^ investigated potential risk factors for elevated levels of COVID-19-related concerns in nurses. Among a sample of 894 Italian nurses, Galletta et al.^32^ found that COVID-19-related worries were typically associated with caring for patients who died of COVID-19, experiencing a high workload, and watching colleagues crying at work. Expanding on this, several authors propose that with increasing age, nurses are at a greater risk for COVID-19-related worries compared to younger nurses.^32,36^ In addition, single and divorced nurses are at a greater risk to COVID-19–related worries compared to nurses with a different marital status.^32^

In a cross-sectional examination of employed individuals in Japan, it was observed that exposure to information sources such as television and the internet correlated with an increased level of fear and worry about COVID-19 infection.^37^ Conversely, findings from an extensive survey conducted in China in 2020 revealed that individuals with higher socioeconomic status were better equipped to handle the challenges posed by COVID-19 and that a heightened sense of community served as a protective factor against worry.^38^ Similarly, within a substantial Norwegian sample, factors such as living alone, economic challenges, pre-existing worry, and prior mental health issues were identified as contributors to elevated distress scores and diminished satisfaction with life.^39^

## Purpose

This study aimed to investigate the extended mental health consequences of the COVID-19 crisis among nurses in South Africa. The study was conducted following the peak of the COVID-19 pandemic, with data collection taking place between April 2022 and May 2023. During this period, while the COVID-19 infection rates in South African hospitals reflected a gradual recovery, ongoing challenges remained. Despite improvements in vaccination coverage, with nearly 50% of the population vaccinated by early 2022, disparities in vaccination rates among younger populations and across provinces contributed to intermittent surges in infections, continuing to strain hospital resources, albeit to a lesser extent than during earlier waves.^40,41^ Additionally, we recognise that the acute mental health challenges faced by HCWs during the pandemic may have long-lasting psychological impacts.^42^ By examining the ongoing psychological distress in this critical workforce, we seek to contribute to the development of informed policy measures and targeted interventions that address the specific mental health needs of nurses in South Africa.

## Methods

### Participants and Procedure

In this cross-sectional study, participants comprised a convenience sample of registered nurses (*N* = 264) working in four South African hospitals located in the Western Cape province of South Africa: Helderberg, TC Newman, Stellenbosch, and Tygerberg. Notices were placed at the hospitals, inviting nurses to contact the study team through a designated WhatsApp number. Participants who contacted the team were sent a link via WhatsApp to an online consent form and questionnaire. The platform utilized for the online survey was Google Forms. For those who preferred to complete a paper version of the survey, arrangements were made to complete the survey on-site at the hospitals. The paper surveys were securely collected by M.J. and L.S. Participation was voluntary, and while confidentiality was maintained, complete anonymity could not be guaranteed due to the use of WhatsApp for recruitment.

Participants provided informed consent electronically via a checkbox option at the beginning of the online survey. For those completing the paper version, written consent forms were provided and signed before they completed the questionnaire. The average time for completing the survey was approximately 20 min. Participants were compensated with an R100 grocery voucher for Shoprite via text. Data were collected between April 2022 and May 2023.

### Instruments

Participants completed a brief demographic survey, the Fear of COVID-19 Scale (FCV-19S),^43^ the Perceived Vulnerability to Disease Questionnaire (PVD-Q),^44^ the Satisfaction with Life Scale (SWLS),^45^ the Beck Hopelessness Scale (BHS),^46^ the Beck Anxiety Inventory (BAI),^47^ the Post-Traumatic Stress Disorder-Checklist for DSM-5 (PCL-5),^48^ the Alcohol Use Identification Test (AUDIT),^49^ the Centre for Epidemiological Studies Depression Scale-Revised (CESD-R),^50^ and a visual analogue scale (VAS) that asked participants to indicate their level of worry about infection with COVID-19, worry and concern about infecting others, worry about hospitalization, and fear of death.

#### Fear of COVID-19

The FCV-19S comprises seven items designed to measure the emotional fear response associated with COVID-19. Participants’ responses were assessed on a 5-point Likert scale, ranging from 1 (*Strongly Disagree*) to 5 (*Strongly Agree*). A satisfactory level of internal consistency has been reported (α = 0.82).^43^ Comparable reliability coefficients exceeding 0.80 have been reported in studies conducted in diverse geographical contexts such as Israel, New Zealand, and Brazil.^51–53^ The reliability and unidimensional structure of the instrument have been substantiated in South African studies.^54^

#### Perceived vulnerability to disease

The PVD-Q comprises 15 items designed to evaluate individual beliefs regarding susceptibility to infectious diseases. Participants responded on a 7-point Likert scale, ranging from *Strongly Disagree* (1) to *Strongly Agree* (7). In an extensive psychometric examination, Duncan et al.^44^ offered internal consistency estimates of 0.87 and 0.74. Robust internal consistency reliability for the PVD-Q has been documented in additional investigations.^55^

#### Satisfaction with life

The SWLS comprises five items, each rated on a 7-point scale, ranging from *Strongly Agree* (7) to *Strongly Disagree* (1). Elevated scores on this scale signify heightened levels of life satisfaction. Pavot and Diener^56^ have reported robust estimates of internal consistency (α > 0.75). A reliability estimate of 0.89 has been reported in the context of South Africa.^57^

#### Hopelessness

The BHS measures the extent to which respondents’ cognitive schema is linked to pessimistic expectations for future events. Comprising 20 items, respondents were required to indicate the veracity of each statement as either “true” or “false.” Beck et al.^46^ indicated a robust internal consistency of 0.93 for the BHS. An alpha coefficient of 0.86 has been reported in the South African context.^58^

#### Anxiety

The BAI comprises 21 items that measure somatic and cognitive aspects of anxiety. Items are rated on a 4-point Likert scale ranging from 1 (*Not at all*) to 4 (*Severely, it bothered me a lot*). Robust internal consistency has been established for the BAI, with alpha coefficients exceeding 0.90 reported in diverse countries such as Korea^59^ and the United States.^60^ When utilized in the South African context, the BAI similarly has exhibited satisfactory internal consistency reliability.^61^

#### Post-traumatic stress

The PCL-5 consists of 20 items to assess the 20 symptoms of PTSD outlined in the DSM-5.^42^ The PCL-5 is utilized to screen individuals from a variety of settings to identify probable PTSD.^48,62,63^ Among Filipino migrant workers (*n* = 131), the PCL-5 scores demonstrated strong internal consistency (α = 0.95) and moderate test-retest reliability (ρ = 0.61, *P* < .001).^63^ In South Africa, Kagee et al.^64^ found that the PCL-5 had optimal sensitivity (0.88) and specificity (0.88) to measure for PTSD, with a suggested cut-off of 32.

#### Alcohol use

The AUDIT comprises 10 items that assess potentially harmful alcohol consumption and symptoms of alcohol dependence. Items are rated on a 4-point Likert scale ranging from 0 to 4. The AUDIT has demonstrated excellent psychometric properties in a number of studies.^65,66^ In South Africa, an alpha coefficient of 0.89 has been reported for the AUDIT.^67^

#### Depression

The CESD-R comprises 20 items that measure nine groups of depression symptoms as outlined in the DSM-5. Items are rated on a 4-point Likert scale ranging from 0 to 4. The CESD-R exhibits favorable psychometric characteristics, demonstrating strong internal consistency as well as effective convergent and divergent validity.^68^ The CESD-R has been utilized in South African studies, with good effect.^69,70^

### Data Analysis

All statistical analyses were conducted using IBM SPSS for Windows version 28 (IBM Corp., Armonk, NY, USA). Following the guideline of 10-15 participants per predictor variable, our sample of 264 exceeded the minimum target of 200. Pearson’s *r* correlation was used to examine relationships between continuous variables, while descriptive statistics summarized demographic and psychological well-being data. As the data were not normally distributed, the Mann-Whitney *U* and Kruskal-Wallis *H* tests were used to explore differences by gender, education, employment status, marital status, and working sector. Employment and student status were treated as mutually exclusive categories. Post-hoc Dunn-Bonferroni tests were applied for pairwise comparisons, with 95% confidence intervals reported.

### Ethical Considerations

Ethical approval was obtained from Stellenbosch University’s Health Research Ethics Committee (Reference number: N21/05/012-COVID-19). Prior to their participation, respondents were required to provide written informed consent on the landing page of the survey. Participants were assured of the voluntary nature of their involvement in the study as well as of their anonymity. Participants were provided with the contact details of free counselling services, in the event of experiencing distress as a consequence of completing the survey.

## Results

The intercorrelations between variables, descriptive statistics, and reliability are reported in Table 1. The sample consisted mostly of married or partnered (54%) women (82%) working in the public sector (76%). The majority of the sample (88%) had completed post-school training (e.g., university/college) and were employed on a full-time basis (90%). The mean age of the sample was 34.4 (*SD* = 7.9).

**Table 1.** Intercorrelations Between Variables, Descriptive Statistics, and Reliabilities of Study Variables (N = 264).

Variables	1	2	3	4	5	6	7	8	9	10
1. Age	—									
2. Fear of Covid19	−0.23**	—								
3. Anxiety	−0.23**	0.51**	—							
4. PVD-Q	0.02	0.39**	0.21**	—						
5. Hopelessness	0.14*	0.03	−0.36**	−0.04*	—					
6. Life satisfaction	0.16*	0.15*	−0.20**	0.07	0.35**	—				
7. PTSD	−0.22**	0.51**	0.57**	0.24**	−0.27**	−0.01	—			
8. Alcohol use	−0.32**	0.27**	0.55**	0.04	−0.35**	−0.06	0.32**	—		
9. Worries	0.05	0.53**	0.21**	0.45**	−0.01	0.07	0.30**	0.08	—	
10. Depression	−0.19**	0.34**	0.80**	0.21**	−0.49**	−0.20**	0.55**	0.50**	0.15*	—
Mean	34.4	20.22	18.04	60.13	34.51	27.17	21.90	17.21	26.78	20.50
*SD*	8.0	8.56	14.81	12.22	4.81	18.61	7.73	7.25	11.04	15.68
Cronbach’s alpha	—	0.94	0.96	0.70	0.88	0.92	0.96	0.90	0.91	0.95

Abbreviations: PTSD: post-traumatic stress disorder; PVD-Q: perceived vulnerability to disease questionnaire; SD: standard deviation.

* *P* < .05. ^**^P < .01.

The reliabilities were generally high, and all exceeded 0.83 (α = 0.84-0.96), with the exception of the PVD-Q (α = 0.70), which was considered acceptable. Table 1 also shows that age was significantly positively associated with hopelessness (*r*_264_ = 0.14, *P* < .05, 95% CI [0.02, 0.26]) and life satisfaction (*r*_264_ = 0.16, *P* < .05, 95% CI [0.04, 0.27]). Age was also significantly negatively associated with fear of COVID (*r*_264_ = −0.23, *P* < .001, 95% CI [−0.34, −0.11]), anxiety (*r*_264_ = −0.23, *P* < .001, 95% CI [−0.34, −0.11]), PTSD (*r*_264_ = −0.22, *P* < .001, 95% CI [−0.33, −0.10]), alcohol use (*r*_264_ = −0.32, *P* < .001, 95% CI [−0.42, −0.20]) and depression (*r_264_* = −0.19, *P* < .01, 95% CI [−0.30, −0.07]). However, in accordance with Cohen’s guidelines as outlined by Gignac and Szodorai,^71^ all the obtained correlation coefficients represent a small effect size, with the exception of alcohol use which may be considered a moderate effect size.

Mann–Whitney *U* tests were conducted to examine differences in psychological well-being and COVID-19-related distress scores according to gender (Supplemental Table 1). The results showed that men demonstrated higher levels of alcohol use compared to women. The mean rank for men was 153.34, and the mean rank for women was 127.36. This difference was statistically significant (*U* = 4073.0, *Z* = −2.130, *P* = .033), indicating that men had significantly higher levels of alcohol use than women.

The Kruskal–Wallis H test was conducted to examine the differences in nurses’ psychological wellbeing and COVID-19-related distress according to employment type (see Table 2 in Supplemental Material). Results reflect that there were differences between the four employment types (full-time, part-time, unemployed, student) in terms of hopelessness (*H*_(3)_ = 9.524, *P* = .02), life satisfaction (*H*_(3)_ = 19.132, *P* < .001), COVID-19-related worries (*H*_(3)_ = 11.077, *P* = .01), and depression (*H*_(3)_ = 11.136, *P* = .01). Post-hoc analyses using Dunn-Bonferroni tests indicated that nurses with full-time employment reported lower levels of depression (adjusted *P* < .05), but higher COVID-19-related worries (adjusted *P* < .05) than unemployed nurses. Further post-hoc analysis using Dunn-Bonferroni tests indicated that nurses with part-time employment reported higher COVID-19-related worries (adjusted *P* < .01) than unemployed nurses. In addition, nurses in full-time employment indicated higher life satisfaction (adjusted *P* < .05) than nurses in part-time employment. There were no other differences between employment types.

Table 3 in the Supplemental Material shows that there were differences between the three education levels (attended high school, but did not complete; attended post-school [university/college], but did not complete; attended post-school, completed) in terms of COVID-19-related worries (*H*_(2)_ = 10.448, *P* = .005), anxiety (*H*_(2)_ = 10.584, *P* = .005), hopelessness (*H*_(2)_ = 22.001, *P* < .001), life satisfaction (*H*_(2)_ = 6.321, *P* = .04), depression (*H*_(2)_ = 10.990, *P* = .004) and alcohol use (*H*_(2)_ = 16.911, *P* < .001). Post-hoc analysis using Dunn-Bonferroni tests indicated that nurses who completed post-school education (e.g., university/college) reported lower levels of depression (adjusted *P* < .05) and alcohol use (adjusted *P* < .05), but higher levels of COVID-19-related worries (adjusted *P* < .05) than nurses who attended high school but did not complete. Further post-hoc analysis using Dunn-Bonferroni tests indicated that nurses who completed post-school education (e.g., university/college) reported lower levels of anxiety (adjusted *P* < .05), alcohol use (adjusted *P* < .01), but higher levels of hopelessness (adjusted *P* < .001), compared to nurses who attended post-school education, but did not complete. There were no other differences between level of education attained.

Table 2 shows that there were differences between the four categories of marital status (married/partnered, separated, divorced, single) in terms of fear of COVID-19 (*H*_(3)_ = 10.870, *P* = .01), anxiety (*H*_(3)_ = 13.364, *P* < .001), COVID-19-related worries (*H*_(3)_ = 8.196, *P* = .04), and depression (*H*_(3)_ = 9.403, *P* = .02). Post-hoc analysis using Dunn-Bonferroni tests indicated that nurses who are married or living with a partner reported lower levels of anxiety (adjusted *P* < .05) compared to nurses who had never been married.

**Table 2.** Kruskal–Wallis Test Results for Marital Status (*N* = 264).

Variable	Marital status	*n*	Mean rank	*H-*value	*P* (adjusted for ties)
Fear of COVID-19	Married/Partnered	143	119.30	10.870	.012
	Separated	15	149.50		
	Divorced	13	172.27		
	Never married	93	144.49		
Anxiety	Married/Partnered	143	117.77	13.364	< .001
	Separated	15	171.67		
	Divorced	13	158.31		
	Never married	93	145.23		
PVD-Q	Married/Partnered	143	136.43	4.139	.25
	Separated	15	147.87		
	Divorced	13	138.73		
	Never married	93	123.11		
Hopelessness	Married/Partnered	143	146.49	4.552	.21
	Separated	15	142.67		
	Divorced	13	122.73		
	Never married	93	110.72		
Life satisfaction	Married/Partnered	143	135.34	.700	.87
	Separated	15	138.30		
	Divorced	13	130.62		
	Never married	93	127.46		
PTSD	Married/Partnered	143	127.44	1.444	.70
	Separated	15	142.57		
	Divorced	13	140.96		
	Never married	93	137.47		
Alcohol use	Married/Partnered	143	122.21	7.584	.06
	Separated	15	162.13		
	Divorced	13	122.69		
	Never married	93	144.91		
Worries	Married/Partnered	143	139.24	8.196	.042
	Separated	15	113.53		
	Divorced	13	171.96		
	Never married	93	119.68		
Depression	Married/Partnered	143	119.81	9.403	.024
	Separated	15	163.30		
	Divorced	13	141.00		
	Never married	93	145.85		

Abbreviations: PTSD: post-traumatic stress disorder; PVD-Q: perceived vulnerability to disease questionnaire.

A Mann–Whitney *U* test showed that nurses working in the private sector reported significantly higher levels of alcohol use (*U* = 5886.500, *P* = .048, *r* = −0.13) and PTSD (*U* = 6183.500, *P* = .008, *r* = −0.17) than those nurses working in the public sector (see Table 4 in Supplemental Material). Both results suggest a small to moderate negative effect size.

## Discussion

This study was conducted following the peak of the COVID-19 pandemic, with data gathered from April 2022 to May 2023. Given that that immediate threat of the pandemic had passed, the objective of this study was to investigate the extended mental health impact of the COVID-19 crisis among nurses in South Africa. Our data showed that overall, nurses reported high levels of anxiety, depression, PTSD, and alcohol consumption. While at least 50% presented with severe anxiety, 29% and 21% presented with moderate and mild anxiety levels, respectively. Similarly, 56% screened positive for depression on the CESD-R, and 26% screened positive for PTSD symptoms on the PCL-5. Concerningly, our results indicated that 52% of nurses reported hazardous levels of alcohol consumption, while 48% demonstrated levels of alcohol dependence. Overall, our findings surpassed those outlined in local studies focusing on HCWs as a broad group.^72^ Interestingly, however, our findings were lower for PTSD than those proposed by local authors Engelbrecht et al.^20^ who found that 44% of nurses screened positive for PTSD, as measured on the Impact of Events Scale-Revised. Overall, our results highlight the profound impact that the COVID-19 pandemic has had on the mental health of nursing staff in South Africa.

We found a negative correlation between age and adverse mental health outcomes among nurses. Specifically, older nurses exhibited reduced levels of fear of COVID-19, anxiety, PTSD, alcohol use, and depression compared to younger nurses. Lower perceived distress among older nurses is likely attributable to heightened levels of maturity and accrued work experience, as mirrored in extant literature.^32,36,73^ On the contrary, older nurses presented with higher levels of hopelessness and life satisfaction, compared to their younger counterparts. The results might suggest that while older nurses may feel weary or less hopeful about the future, their satisfaction with what they have achieved in life and their ability to find meaning in their work or personal lives remain high.^74^

Contrary to expectations, we found no significant gender differences in psychological well-being and COVID-19-related distress among nurses, with the notable exception of alcohol consumption. Specifically, male nurses demonstrated significantly higher levels of alcohol use compared to their female counterparts. This finding diverges from existing research, which has often reported heightened adverse psychological symptoms among female nurses relative to males.^75,76^ Consistent with broader literature, however, men generally consume alcohol more frequently and in larger quantities than women, a trend that extends to the nursing profession.^77^

Significant differences in psychological wellbeing and COVID-19-related distress among nurses emerged for employment type. Specifically, full-time nurses reported lower levels of depression compared to unemployed nurses. Likewise, part-time nurses demonstrated increased life satisfaction compared to unemployed nurses. The reduced depression levels in full-time nurses, and heightened life satisfaction among part-time nurses, may be attributable to factors such as job stability, financial security, and a sense of professional purpose associated with consistent employment, especially in a global health crisis.^77^ Not surprisingly, full-time and part-time nurses indicated higher COVID-19-related worries compared to unemployed nurses, likely stemming from increased exposure to COVID-19-infected patients, and thus increased worries about becoming infected and infecting loved ones.^32,33^

We found a negative correlation between education levels attained and mental health outcomes among nurses. Specifically, those with post-school education (e.g., university/college) demonstrated reduced levels of depression and alcohol use in contrast to nurses who attended high school but did not complete their education. Additionally, nurses who completed their post-school education (e.g., university/college) exhibited reduced levels of anxiety and alcohol use compared to their counterparts who attended, but did not complete, their post-school education. These findings align with existing research^77^ suggesting that higher education levels among nurses are associated with a reduced likelihood of developing adverse mental health outcomes.

Marital status was associated with differences in fear of COVID-19, anxiety, hopelessness, COVID-19-related worries, and depression. Specifically, nurses who were married or living with a partner reported reduced levels of anxiety compared to their unmarried counterparts, mirroring extant literature.^78^ The results emphasize social support and the presence of a stable relationship as being a significant protective factor to the mental wellbeing of nurses amid a public health crisis.

South Africa’s health system is characterized by a two-tiered structure, comprising the public (government-run) and private health sectors.^8^ Unfortunately, chronic underfunding and mismanagement of the public healthcare sector, serving around 80% of the population,^8^ have resulted in major health system challenges, inclusive of poor staff attitudes, extended waiting periods, substandard facility hygiene, medication shortages, inadequate infection control measures, and compromised safety and security for both staff and patients.^79^ In this study, differences were found in mental health outcomes among nurses between the public and private sectors. Our results show that private sector nurses reported higher alcohol use and post-traumatic stress than public sector nurses. This may be due to differences in occupational environments. Public sector nurses in South Africa, often exposed to high-stress, resource-limited settings, may develop resilience or habituation to stress. In contrast, private sector nurses face stressors like performance metrics and financial targets, which may increase stress and lead to maladaptive coping behaviors such as elevated alcohol use. Further investigations into these sector-specific factors are thus necessary to inform targeted interventions.

These findings have significant implications for understanding the extended mental health impact of the COVID-19 pandemic among nurses. The persistence of high levels of anxiety, depression, PTSD, and alcohol consumption even after the pandemic’s peak highlights the enduring nature of these effects. Moreover, the study identifies critical demographic and occupational factors such as age, employment type, education level, and marital status, which influence mental health outcomes, offering valuable insights for targeted interventions. Furthermore, the results contribute to advancing the literature by demonstrating that younger nurses, those with lower educational attainment, and public sector nurses may be particularly vulnerable to adverse outcomes, reinforcing the need for differentiated mental health strategies. These findings also emphasize the protective role of education, social support, and employment stability, aligning with broader research on the positive effects of social support and economic stability on mental well-being.^23,80^ The implications of this study are far-reaching, advocating for ongoing mental health interventions and tailored support systems to mitigate the pandemic’s extended psychological impact among HCWs, and particularly among those in underrepresented and under resourced regions.

### Strengths and Limitations

This study exhibits various strengths and limitations. Notable strengths involve the study’s novel contribution to understanding the extended mental health impact of the pandemic among a susceptible workforce. Nevertheless, this study confronts limitations, which impede the generalizability of the findings. First, the cross-sectional data were obtained from four out of more than 600 hospitals in South Africa, with considerable variability in access to resources across provinces and between hospitals. Second, participants voluntarily opted to complete the survey, and thus self-selection bias was introduced. Third, the data utilized in this study are cross-sectional and reliant on self-reporting, thereby limiting the ability to make strong causal inferences.

## Conclusion

Our findings, in alignment with extant research, indicate poor psychological outcomes among nurses in South Africa amid the pandemic. Significantly, our data collection spanned the years 2022 to 2023. Thus, even with some time having passed and the lifting of stringent lockdown measures, it is apparent that nurses were still grappling with mental health challenges and psychological distress, underscoring the need for improved and ongoing psychological support.

## Supplemental Material

sj-pdf-1-wjn-10.1177_01939459251316049 – Supplemental material for Unmasking Distress: An Analysis of COVID-19’s Mental Health Impact on Nurses in South Africa Supplemental material, sj-pdf-1-wjn-10.1177_01939459251316049 for Unmasking Distress: An Analysis of COVID-19’s Mental Health Impact on Nurses in South Africa by Phillipa Haine, Ashraf Kagee, Bronwyne Coetzee, Marnus Janse Van Vuuren and Lindokuhle Shongwe in Western Journal of Nursing Research
