# Supplementary material for: Unmasking Distress: An Analysis of COVID-19’s Mental Health Impact on Nurses in South Africa
Source: West J Nurs Res. 2025 Feb 3;47(4):241–51. doi: 10.1177/01939459251316049 (PMC11909779; doi:10.1177/01939459251316049)
Supplement: sj-pdf-1-wjn-10.1177_01939459251316049 – Supplemental material for Unmasking Distress: An Analysis of COVID-19’s Mental Health Impact on Nurses in South Africa [file sj-pdf-1-wjn-10.1177_01939459251316049.pdf]

Supplementary Material

Table 1

Mann-Whitney U test results for gender

Variable	Gender	*n*	Sum of Ranks	*U*	Sig. (adjusted for ties)
Fear of COVID-19	Women	172	27965.00	4590.00	.25
	Men	67	6751.00		
Anxiety	Women	172	28123.50	4687.50	.41
	Men	67	6592.50		
PVDS	Women	172	28341.50	4905.50	.72
	Men	67	6374.50		
Hopelessness	Women	172	29051.00	4537.00	.25
	Men	67	5665.00		
Life Satisfaction	Women	172	27933.00	4497.00	.22
	Men	67	6783.00		
PTSD	Women	172	28182.00	4746.00	.49
	Men	67	6534.00		
Alcohol Use	Women	172	27509.00	4073.00	.033*
	Men	67	7207.00		
Worries	Women	172	27604.50	4168.50	.05
	Men	67	7111.50		
Depression	Women	172	28194.00	4758.00	.50
	Men	67	6522.00		

Note: PVDS = Perceived Vulnerability to Disease. $^{**}p < .01$, $^{*}p <. 05$

Table 2

Kruskal-Wallis test results for employment status

Variable	Employment	*n*	Mean Rank	*H*-Value	Sig. (adjusted for ties)
Fear of COVID-19	Full-time	238	132.48	1.450	.69
	Part-time	20	142.48		
	Unemployed	4	96.75		
	Student	2	106.75		
Anxiety	Full-time	238	130.15	7.579	.06
	Part-time	20	136.25		
	Unemployed	4	196.75		
	Student	2	246.50		
PVDS	Full-time	238	135.04	3.597	.31
	Part-time	20	105.05		
	Unemployed	4	105.38		
	Student	2	158.75		
Hopelessness	Full-time	238	135.71	9.524	.023*
	Part-time	20	121.30		
	Unemployed	4	53.25		
	Student	2	21.00		
Life Satisfaction	Full-time	238	138.84	19.132	<.001**
	Part-time	20	86.78		
	Unemployed	4	42.75		
	Student	2	15.00		
PTSD	Full-time	238	129.69	3.386	.34
	Part-time	20	160.13		
	Unemployed	4	147.00		
	Student	2	162.00		
Alcohol Use	Full-time	238	132.73	7.169	.07
	Part-time	20	111.73		
	Unemployed	4	223.13		
	Student	2	132.00		
Worries	Full-time	238	133.71	11.077	.011*
	Part-time	20	146.05		
	Unemployed	4	13.63		
	Student	2	90.50		
Depression	Full-time	238	130.36	11.136	.011*
	Part-time	20	127.20		
	Unemployed	4	237.13		
	Student	2	231.00		

Note: PVDS = Perceived Vulnerability to Disease. $^{**}p < .01$, $^{*}p <. 05$

Table 3

Kruskal-Wallis test results for level of education

Variable	Education	*n*	Mean Rank	*H*-Value	Sig. (adjusted for ties)
Fear of COVID-19	Post-school	232	132.18	.328	.85
	Post-school DNC	28			
	High School DNC	4	115.50		
Anxiety	Post-school	232	127.04	10.584	.005**
	Post-school DNC	28			
	High School DNC	4	203.75		
PVDS	Post-school	232	133.79	1.985	.37
	Post-school DNC	28			
	High School DNC	4	80.38		
Hopelessness	Post-school	232	140.59	22.001	<.001**
	Post-school DNC	28			
	High School DNC	4	55.63		
Life Satisfaction	Post-school	232	136.82	6.321	.042*
	Post-school DNC	28			
	High School DNC	4	85.88		
PTSD	Post-school	232	130.94	.919	.63
	Post-school DNC	28			
	High School DNC	4	131.25		
Alcohol Use	Post-school	232	125.80	16.911	<.001**
	Post-school DNC	28			
	High School DNC	4	232.00		
Worries	Post-school	232	136.88	10.448	.005**
	Post-school DNC	28			
	High School DNC	4	28.75		
Depression	Post-school	232	127.37	10.990	.004**
	Post-school DNC	28			
	High School DNC	4	224.38		

Note: PVDS = Perceived Vulnerability to Disease. DNC = Did Not Complete. $^{**}p < .01$, $^{*}p < .05$

Table 4

Mann-Whitney U test results for working sector

Variable	Education	*n*	Sum of Ranks	*U*	Sig. (adjusted for ties)
Fear of COVID-19	Government	176	114.74	5413.500	.96
	Private	57	123.97		
Anxiety	Government	176	112.12	5875.000	.05
	Private	57	132.07		
PVDS	Government	176	113.56	5627.500	.17
	Private	57	127.73		
Hopelessness	Government	176	119.36	4600.000	.35
	Private	57	109.70		
Life Satisfaction	Government	176	120.97	4318.000	.11
	Private	57	104.75		
PTSD	Government	176	110.37	6183.500	.008**
	Private	57	137.48		
Alcohol Use	Government	176	112.05	5886.500	.048*
	Private	57	132.27		
Worries	Government	176	116.88	5038.000	.37
	Private	57	117.39		
Depression	Government	176	114.70	5421.100	.36
	Private	57	124.11		

Note: PVDS = Perceived Vulnerability to Disease. $^{**}p < .01$, $^{*}p <. 05$
